# Associations between health literacy and preventive health behaviors among older adults: findings from the health and retirement study

**DOI:** 10.1186/s12889-016-3267-7

**Published:** 2016-07-19

**Authors:** Dena M. Fernandez, Janet L. Larson, Brian J. Zikmund-Fisher

**Affiliations:** Department of Internal Medicine, Division of General Medicine, University of Michigan, 1500 E. Medical Center Drive, Ann Arbor, MI 48109-2029 USA; Department of Health Behavior and Biological Sciences, University of Michigan School of Nursing, 400 North Ingalls Building, Ann Arbor, MI 48109-5482 USA; Department of Health Behavior & Health Education, School of Public Health, University of Michigan, 1415 Washington Heights, Ann Arbor, MI 48109-2029 USA; Institute for Healthcare Policy and Innovation, Center for Bioethics and Social Sciences in Medicine, 2800 Plymouth Road, Ann Arbor, MI 48109-2800 USA

**Keywords:** Health literacy, Perceptions, Health behaviors, Preventive health behaviors, Mammography, Perceived control, Tobacco, Physical activity, Discrimination

## Abstract

**Background:**

While the association between inadequate health literacy and adverse health outcomes has been well documented, less is known about the impact of health literacy on health perceptions, such as perceptions of control over health, and preventive health behaviors.

**Methods:**

We identified a subsample of participants (*N* = 707) from the Health and Retirement Study (HRS), a nationally representative sample of older adults, who participated in health literacy testing. Self-reported health literacy was measured with a literacy screening question, and objective health literacy with a summed score of items from the Test of Functional Health Literacy. We compared answers on these items to those related to participation in health behaviors such as cancer screening, exercise, and tobacco use, as well as self-referencing health beliefs.

**Results:**

In logistic regression models adjusted for gender, education, race, and age, participants with adequate self-reported health literacy (compared to poorer levels of health literacy) had greater odds of participation in mammography within the last 2 years (Odds ratio [OR] = 2.215, *p* = 0.01) and participation in moderate exercise two or more times per week (OR = 1.512, *p* = 0.03). Participants with adequate objective health literacy had reduced odds of participation in monthly breast self-exams (OR = 0.369, *p* = 0.004) and reduced odds of current tobacco use (OR = 0.456, *p* = 0.03). In adjusted linear regression analyses, self-reported health literacy made a small but significant contribution to explaining perceived control of health (β 0.151, *p* = <0.001) and perceived social standing (β 0.112, *p* = 0.002).

**Conclusion:**

In a subsample of older adult participants of the HRS, measures of health literacy were positively related to several health promoting behaviors and health-related beliefs and non-use of breast self-exams, a screening behavior of questionable benefit. These relationships varied however, between self-reported and objectively-measured health literacy. Further investigation into the specific mechanisms that lead higher literacy people to pursue health promoting actions appears clearly warranted.

## Background

Health literacy, the capacity of individuals to obtain and understand health information needed to make health decisions, is recognized by the Institute of Medicine and the federal government as fundamental to quality health care [1–3]. However, findings from the National Assessment of Adult Literacy (a nationally representative assessment of literacy among adults in the United States) suggest that health literacy is limited in a third of adults and in a markedly larger portion of older adults [4, 5]. Reports from health literacy research estimate rates of limited health literacy in 33–60 % of adults in outpatient and inpatient settings [6–11]. Limited health literacy has consistently been shown to be a strong predictor of poorer health outcomes [12]. Individuals with limited health literacy are prone to greater use of emergency services [13–15], higher rates of hospitalization [16, 17], and higher rates of mortality [18, 19]. Limited health literacy is also associated with less use of preventive services [14, 20], and poorer adherence to medication regimens [21, 22].

The relationship between health literacy and health outcomes is well established, but the mechanisms underlying this relationship are not yet fully understood. Commonly cited theoretical models suggest that health literacy influences health outcomes at least partially through its effect on patients’ self-efficacy, knowledge, and health behaviors [23–25] as well as their health-related perceptions, health-related experiences, and familiarity with health concepts [26, 27]. However, these models have not yet been tested in full, and further work is needed to empirically validate the proposed relationships in these frameworks [28]. The purpose of this study is to examine relationships between health literacy and health perceptions, and between health literacy and health behaviors in a sample of older adults, a population segment growing in size with a greater rate of chronic conditions, utilization of medical services, and limited health literacy than other age groups.

## Methods

### Study design and data sources

We conducted a secondary analysis of cross-sectional data from supplemental questionnaires administered as part of the Health and Retirement Study (HRS). The HRS is a biennial longitudinal interview survey of U.S. adults over the age of 50 sponsored by the National Institute on Aging and conducted by the Institute for Social Research (ISR) at the University of Michigan ([29, 30], The Health and Retirement Study). Employing a multi-stage area probability sampling design, HRS researchers began collecting data on issues of aging, health, and retirement in 1992. Since then, core interviews have been repeated on the original sample, along with newly added cohorts, every two years.

To complement the core surveys, supplemental questionnaires have been administered to both random and purposefully selected respondents. Internet based surveys are one type of supplemental questionnaire for which HRS participants who report regular use of the internet in the core survey are eligible. Internet based surveys are conducted in the alternate years and cover topics including health behaviors and health-related knowledge. In the HRS 2009 Internet Survey, health literacy was additionally assessed; however, only a random subsample was selected to undergo health literacy testing with both reading comprehension and numeracy items. The Psychosocial and Lifestyle Questionnaire (PLQ) is a supplemental questionnaire that was designed to elicit participants’ views of their life, health, and well-being. This questionnaire is given to a random 50 % of the core participants who are interviewed face to face; the questionnaire is left behind with them to complete and mail-in.

### Sample

In the present study, data from the HRS 2009 Internet Survey were linked to the HRS 2008 Core Survey to obtain a more comprehensive list of covariates on the same respondents. Household and individual identification numbers were used in order to distinguish persons who participated in both waves. For analysis purposes, we focus on those individuals who were administered the PLQ and offered all of the health literacy questions in the internet survey. Of 5,742 HRS respondents invited to participate in the internet survey, 4,433 participated (yielding a response rate of 77.2 %). Of the 4,433 initial participants that started the internet survey, a total of 4,351 completed the survey. The PLQ was completed by 1,902 of those 4,351, and of those, 707 underwent health literacy testing with both reading comprehension and numeracy items.

### Variables and measurements

#### Health literacy

Health literacy was measured in the HRS using items from the Test of Functional Health Literacy (TOFHLA) [31] and a literacy screener [32]. The TOFHLA is a measure of an individual’s ability to read and understand two passages, and to understand and use quantitative information [31]. The HRS employed seven TOFHLA reading comprehension items and seven TOFHLA numeric items. In the present study, respondents are given one point for each correct answer. The data were not normally distributed, so we followed the procedures of Parker et al. [31] and labeled participants with < 60 correct as having inadequate health literacy, those with 60–75 correct as having marginal health literacy, and those with greater than 75 % correct as having adequate health literacy. We dichotomized responses as either adequate or inadequate by merging inadequate and marginal categories [33–35]. Health literacy determined by these TOFHLA items is termed from here on as objective health literacy.

The Chew health literacy screener is a single item screening question that asks “How confident are you filling out medical forms by yourself?” [32]. This screening question has been shown to be a valid and reliable predictor of functional health literacy [32, 36]. Possible responses are on a Likert style scale and range from 0–4, zero being “extremely” and 4 being “not at all.” We also dichotomized this variable based on the recommended cut-off of 2 or less to identify adequate health literacy [36, 37]. Health literacy determined by this screener is termed from here on as self-reported health literacy.

### Dependent variables

We selected perceived control over health and perceived healthcare discrimination as measures of health perceptions plausibly related to health literacy based on positive relationships demonstrated in health literacy research between limited health literacy and healthcare-related patient dissatisfaction, reduced participation, and shame [38–40]. We additionally examined the outcome of perceived social standing because of the well-documented relationships between social standing and the incidence and severity of disease and access to care in the U.S. and other countries [41–43]. We assessed perceived control over health with a single item which asks participants to “Think about a 0 to 10 scale where ‘0’ means ‘no control at all’ and ‘10’ means ‘very much control.’ How would you rate the amount of control you have these days over your health?” [44]. We assessed perceived social standing with the MacArthur Scale of Subjective Social Status [45], which asks participants to select a rung on a ladder that represents where they believe they stand in society, with the top representing those who are doing the best (0–10). We assessed perceived healthcare discrimination with a single item that asked “In day-to-day life, how often have any of the following things happened to you?” [You receive poorer service or treatment than other people from doctors or hospitals] (almost every day, at least once a week, a few times a month, a few times a year, less than once a year, never) [46, 47]. We additionally measured perceptions of risks of colon cancer, risks of colon cancer death, and benefits of colon cancer screening with the following three items: “Out of every 100 people, about how many do you think will die of colon cancer?” (0–100), “Out of every 100 people, about how many will be diagnosed with colon cancer at some time in their lives?” (0–100), and “Do you think regular colon cancer screening for people over age 50 does or does not reduce the risk of dying from colon cancer?” (does/does not/don’t know) [48].

We also measured 8 specific health behaviors: flu immunization, cholesterol testing, mammography, breast self-examination (BSE), prostate exam, current tobacco use, and moderate and vigorous physical activity. Participants were asked if they had participated in flu immunization in the last 2 years, if they had cholesterol screening within the last 2 years, if they currently smoked, and how often they performed moderate and vigorous physical activity (daily, more than once per week, once per week, 1–3 times per month, hardly ever, or never). Female participants were asked if they had a mammogram within the last 2 years and whether they performed BSE monthly (yes/no); male participants were asked whether they had a prostate exam in the last 2 years.

### Analyses

To examine the associations between health literacy and health perceptions and between health literacy and health behaviors, we first conducted bivariate analyses. Chi-squared tests were used for categorical outcomes and the Yates correction for continuity was applied as recommended for each condition in which there were two categories per variable [49]. The Mann–Whitney *U* test was used for the two continuous health perception variables (perceived control over health and perceived social standing). We selected this test because scores were skewed for perceived control over health and perceived social standing.

We conducted multiple regression analyses to evaluate the influence of health literacy on health perceptions and health behaviors. To examine relationships between health literacy and health perceptions, we conducted linear regressions for perceived control of health and perceived social standing. In preliminary analyses we examined normality, linearity, multicollinearity, and homoscedasticity. We checked normal probability plots of the regression standardized residual for normality as well as a scatterplot of the standardized residuals for homoscedasticity and outliers. We also reviewed the correlations tables closely for any strong correlations between the independent variables. We conducted logistic regressions for each categorical health perception variable (including perceived healthcare discrimination and perceived colon cancer risks) and to examine relationships between health literacy and health behaviors, regressing the dichotomous health behaviors (including flu immunization, cholesterol testing, mammography, BSE, prostate exam, current tobacco use, and physical activity) against first objective health literacy and then self-reported health literacy. Each multivariable analysis controlled for the socio-demographic characteristics of gender, education, race, and age because these variables have been previously identified as confounding variables in the relationship between health literacy and health outcomes [4, 50–53].

## Results

### Sample characteristics

Participants in the final sample (*N* = 707) were predominantly white (92), and approximately half were female (57 %). Participants had a mean age of 66, and most were married (81 %). The sample was well educated with almost 95 % of participants having at least a high school education. College educated and post-college educated participants made up 45 and 24 % of the sample respectively. Self-reported health literacy scores ranged from 0–4, with a mean of 0.84 (and standard deviation of 1.02). Objective health literacy scores ranged from 0–14, with a mean score of 12.45 (and standard deviation of 1.72) Table 1.

### Associations of health literacy with health perceptions

In bivariate analyses, there were significant differences between participants with adequate and inadequate self-reported health literacy in perceived control over health, Md = 8 versus Md = 7 respectively, (z = −4.91, *p* = <0.0005), and in perceived social standing, Md = 7 versus Md = 6 respectively, (z = −4.15, *p* = <0.0005). Also, compared to participants with inadequate self-reported health literacy, participants with adequate self-reported health literacy were more likely to correctly respond that colon cancer screening does reduce the risk of dying from colon cancer, 89.1 % versus 81.9 %, ($x$^2^ = 4.94, *p* = 0.026).

In adjusted linear regression analyses that evaluated the influence of self-reported health literacy on perceived control over health and perceived social standing, self-reported health literacy made a statistically significant contribution to explaining perceived control of health (β 0.151, *p* = <0.0005) and perceived social standing (β 0.112, *p* = 0.002), however the R-Square values were weak (0.053 and 0.169 respectively). In adjusted logistic regression models, self-reported health literacy did not significantly influence the likelihood of reporting healthcare discrimination or correctly responding to the colon cancer items (Table 2).

**Table 1:** Characteristics of Participants (*N* = 707)

Characteristic	Mean +/− SD or %
Age (years)	65.80 +/− 9.05
Gender (Female)	57.1
Race/Ethnicity	
White	92.1
Black	5.2
Other^a^	2.7
Hispanic	4.4
Education	
0–8 years	1.0
9–11 years	4.6
12 years	25.3
College	45.2
Post-college	23.9
Marital Status	
Married or Live-in Partner	80.8
Unmarried^b^	19.2
Employment	
Employed	36.4
Unemployed^c^	12.4
Retired	46.8
Homemaker	4.4

Note: Final sample *N* = 707. Valid percentages reported due to missing data. HTN = hypertension; SD = standard deviation

^a^Other race includes American Indian, Alaskan Native, Asian, and Pacific Islander

^b^Unmarried includes separated, divorced, never married, widowed, and one refusal to respond

^c^Unemployed includes unemployed and looking for work, laid off, disabled, sick or other leave, and one refusal to respond

**Table 2:** Multivariate adjusted association of adequate self-reported health literacy with health perceptions and behaviors

Health perceptions	Adjusted^a^ OR^b^	CI	*P*
Perceived control over health	β(SE) 0.759 ± 0.188	βs 0.148	< 0.0005
Perceived social standing	β(SE) 0.460 ± 0.147	βs 0.112	0.002
Perceived healthcare discrimination	0.69	(0.43–1.12)	0.13
Perceptions of colon cancer (correct)			
Perceived risk of diagnosis	1.39	(0.78–2.50)	0.27
Perceived risk of death	1.68	(0.89–3.16)	0.11
Perceived benefit of screen	1.63	(0.97–2.72)	0.064
Health Behaviors			
Flu immunization	0.94	(0.62–1.43)	0.78
Cholesterol testing	1.41	(0.77–2.59)	0.26
Mammography	2.22	(1.21–4.66)	0.010
Self-breast exam	0.66	(0.39–1.13)	0.13
Prostate examination	1.27	(0.68–2.40)	0.46
Current tobacco use	0.55	(0.30–1.01)	0.054
Moderate Physical activity	1.51	(1.03–2.21)	0.033
Vigorous Physical activity	1.21	(0.81–1.80)	0.35

^a^Adjusted for age, gender, race, and education

^b^Logistic regression unless otherwise noted with a β (SE). β; unstandardized slope coefficient. Bs; standardized slope coefficient. SE; Standard Error

Bivariate associations between objective health literacy and health perceptions were not significant. In adjusted linear regression analyses that evaluated the influence of objective health literacy on perceived control over health and perceived social standing, the models were statistically significant but weak, and did not identify objective health literacy as a statistically significant or unique contributor in explaining the rates of perceived control over health or perceived social standing. In adjusted logistic regression models, objective health literacy did not significantly influence the likelihood of reporting healthcare discrimination or correctly responding to the colon cancer items (Table 3).

**Table 3:** Multivariate adjusted association of adequate objective health literacy with health perceptions and behaviors

Health perceptions	Adjusted^a^ OR^b^	CI	P
Perceived control over health	β(SE)–0.14 ± 0.21	βs–0.02	0.52
Perceived social standing	β(SE)–0.12 ± 0.17	βs–0.03	0.47
Perceived healthcare discrimination	1.04	(0.59–1.84)	0.90
Perceptions of colon cancer (correct)			
Risk of diagnosis	1.89	(0.90–3.96)	0.09
Perceived risk of death	1.75	(0.83–3.68)	0.14
Perceived benefit of screen	1.72	(0.98–3.02)	0.06
Health Behaviors			
Flu immunization	0.93	(0.58–1.51)	0.78
Cholesterol testing	1.72	(0.86–3.44)	0.12
Mammography	1.54	(0.74–3.21)	0.25
Breast self-exam	0.37	(0.19–0.73)	0.004
Prostate examination	1.02	(0.50–2.10)	0.95
Current tobacco use	0.46	(0.23–0.91)	0.025
Moderate physical activity	0.94	(0.61–1.46)	0.79
Vigorous physical activity	0.93	(0.59–1.46)	0.75

^a^Adjusted for age, gender, race, and education

^b^Logistic regression unless otherwise noted with a β (SE). β; unstandardized slope coefficient. βs; standardized slope coefficient. SE; Standard Error

### Associations of health literacy with behavior variables

In bivariate analyses, significant associations were demonstrated between self-reported health literacy and mammography, moderate physical activity, and tobacco use. Comparing the two groups, participants with adequate self-reported health literacy were more likely to report having a mammogram within the last 2 years (85 % versus 69 %) $\Big(x$^2^ = 8.67, *p* = 0.003), more likely to report performing moderate physical activity two or more times per week (66 % versus 54 %) $\Big(x$^2^ = 6.76, *p* = 0.009), and less likely to report current tobacco use (8 % versus 14 %) $\Big(x$^2^ = 5.08, *p* = 0.024). In adjusted logistic regression analyses, participants with adequate self-reported health literacy had greater odds (compared to participants with inadequate self-reported health literacy) of participation in mammography (OR = 2.215, *p* = 0.010) and moderate physical activity (OR = 1.512, *p* = 0.033), and a tendency toward lower odds of current tobacco use (OR = 0.550, *p* = 0.054), although this did not reach statistical significance (Table 2).

Bivariate analyses between objective health literacy and health behaviors revealed a significant association between objective health literacy and BSE. Women with adequate objective health literacy were less likely to report monthly BSE compared to those with inadequate objective health literacy (49.4 % versus 72 %) $\Big(x$^2^ = 8.056, *p* = 0.005). In adjusted logistic regression models, women with adequate objective health literacy had reduced odds of reporting participation in monthly BSE (Odds ratio [OR] = 0.369, *p* = 0.004) and reduced odds of reporting current tobacco use (OR = 0.456, *p* = 0.025) (Table 3).

## Discussion

To the best of our knowledge, this secondary analysis is the first study to investigate the relationships between health literacy and health behaviors and perceptions in a subsample of a nationally representative sample of adults. The principal findings include significant positive relationships between self-reported health literacy and health behaviors of mammography and physical activity, and perceptions of social standing and control over health. Additionally, significant inverse relationships were noted between objective health literacy and participation in monthly BSE and current use of tobacco.

Findings from this study confirm those of previous studies by demonstrating that women with inadequate self-reported health literacy were less likely to have had a mammogram in the last 2 years [20, 54–56]. However the negative relationship observed between objective health literacy and monthly BSE practices in the women in this sample, to our knowledge, has not been identified in previous literature. This finding was unexpected, but is possible that women with lower health literacy are utilizing BSE to replace mammography due to barriers accessing health services, which has been identified as especially challenging for those with inadequate health literacy [1]. Alternatively, it is possible that women with higher levels of health literacy are aware of the limitations of BSE, which at this time is actually not recommended as a breast cancer screening method by the World Health Organization [57]. Because mammography is known to be effective in reducing breast cancer mortality, and BSE is not [58], future research is needed to explore the knowledge, beliefs, and breast cancer screening practices of women of all health literacy levels.

Prior research regarding the relationship between health literacy and smoking status is limited and inconsistent. Our results support the findings of Von Wagner et al. [25] that lower health literacy increased the odds of reporting being a current smoker. Our results are not consistent with findings of Baker et al. [18] who found negative associations between health literacy and smoking behaviors, but the relationship lost significance in adjusted analyses. Future research is needed to confirm and describe the relationship between health literacy and tobacco use, and to further explore its underlying mechanisms.

Other important results from the present study include positive relationships between health literacy and perceived social standing. This finding is consistent with those of Van De Heide et al. [59], who examined the health literacy of the general population of the Netherlands using the Dutch data from the European Health Literacy Survey (HLS-EU) (*N* = 925). They demonstrated significant positive relationships between three of four domains of health literacy and perceived social status. Additionally, the findings from qualitative studies of adults with limited health literacy provide theoretical support for a positive relationship between health literacy and perception of social standing by describing the negative self-perceptions held by individuals with inadequate health literacy. In one study, participants with inadequate health literacy described feeling bad about themselves, worthless, or lazy for not having obtained health literacy [39]. In another study, participants with inadequate health literacy revealed worries that they may be seen by others as a bad people or incompetent members of society [60].

This study has limitations that must be considered. The first set of limitations is related to external validity. Although sampling methods employed by the HRS were designed to obtain a nationally representative sample, there was some reading involved in all modes used by the HRS to recruit and collect data, which may have led to self-selection bias (those with the lowest literacy refusing to participate in the HRS). The HRS subsample used for this study was invited from the core sample based on their report of regular internet access and use, which may have led to another recruitment bias. Either of these biases may have contributed to the overrepresentation of more educated adults in this sample.

A second set of limitations is related to the measurement of health literacy. Health literacy as a concept is comprised of not only reading and quantitative ability, but an interaction between knowledge, societal, and cultural influences that are difficult to measure [1]. In fact, experts agree that *all* existing measures of health literacy are inadequate or incomplete [61] and that none comprehensively assess the capacity of an individual [26]. In this study, objective health literacy was measured by performance on a limited number of available TOFHLA items. While the Short Test of Functional Health Literacy in Adults (STOFHLA), which is made up 36 TOFHLA items, has had reliability and validity supported in diverse populations [18, 22, 62], in this study a health literacy index of 14 TOFHLA items was used. This may make comparisons with the TOFHLA or STOFHLA difficult. In addition, the different predictive abilities of the self-report and objective measures demonstrated in this study suggest that they may be assessing related but different constructs. Further studies are needed to closely examine and compare the construct validity of these commonly used measures of health literacy.

Finally, given data limitations, several single item scales were employed in this study. While single literacy screening items have been shown to correlate strongly with well accepted multiple-item health literacy measures [32, 36, 37, 63], other single item variables in this study have unknown validity and utility. Perceived healthcare discrimination was measured with a question from the multiple-item Everyday Discrimination Scale that was modified to inquire about discrimination in the healthcare setting [47]. Further testing for correlation of this item with validated measures of healthcare discrimination (such as the Discrimination in Medical Settings Scale; [64]) is needed to determine the validity of this question.

## Conclusion

The purpose of this study was to examine relationships between health literacy and health behaviors and perceptions in a sample of older adults. Results provided evidence for significant relationships between health literacy and breast screening behaviors, physical activity, and current tobacco use, as well significant relationships between health literacy and perceived control over health and perceived social standing. Future research is needed to further examine the impact of health literacy on these important health behaviors and to describe the psychosocial experience of individuals with inadequate health literacy.

## Abbreviations

BSE, breast self-exam; HRS, health and retirement study; OR, odds ratio; PLQ, psychological and Lifestyle Questionnaire; STOFHLA, short test of functional health literacy in adults; TOFHLA, test of functional health literacy in adults
